# Unravelling the proteomic profile of rice meiocytes during early meiosis

**DOI:** 10.3389/fpls.2014.00356

**Published:** 2014-07-24

**Authors:** Melania Collado-Romero, Enriqueta Alós, Pilar Prieto

**Affiliations:** Plant Breeding Department, Institute for Sustainable Agriculture, Agencia Estatal Consejo Superior de Investigaciones CientíficasCórdoba, Spain

**Keywords:** prophase I, chromosome recognition, chromosome pairing, meiocytes proteome, SKP1 proteins, cytoskeleton, MS/MS

## Abstract

Transfer of genetic traits from wild or related species into cultivated rice is nowadays an important aim in rice breeding. Breeders use genetic crosses to introduce desirable genes from exotic germplasms into cultivated rice varieties. However, in many hybrids there is only a low level of pairing (if existing) and recombination at early meiosis between cultivated rice and wild relative chromosomes. With the objective of getting deeper into the knowledge of the proteins involved in early meiosis, when chromosomes associate correctly in pairs and recombine, the proteome of isolated rice meiocytes has been characterized by nLC-MS/MS at every stage of early meiosis (prophase I). Up to 1316 different proteins have been identified in rice isolated meiocytes in early meiosis, being 422 exclusively identified in early prophase I (leptotene, zygotene, or pachytene). The classification of proteins in functional groups showed that 167 were related to chromatin structure and remodeling, nucleic acid binding, cell-cycle regulation, and cytoskeleton. Moreover, the putative roles of 16 proteins which have not been previously associated to meiosis or were not identified in rice before, are also discussed namely: seven proteins involved in chromosome structure and remodeling, five regulatory proteins [such as SKP1 (OSK), a putative CDK2 like effector], a protein with RNA recognition motifs, a neddylation-related protein, and two microtubule-related proteins. Revealing the proteins involved in early meiotic processes could provide a valuable tool kit to manipulate chromosome associations during meiosis in rice breeding programs. The data have been deposited to the ProteomeXchange with the PXD001058 identifier.

## Introduction

Cultivated rice (*Oryza sativa* L.) is one of the most economically important food crops in the world and a staple food for more than half of the world's population (Khush, 2005). Estimations indicate that rice production should feed 5 billion consumers by 2030, which means a 40% yield increase (Khush, 2005). Hence, rice breeding projects are mostly focused on the satisfaction of the increasing demand of grain. In addition, the improvement of plant adaptation to climate change and extreme hydrological fluctuations, as well as the generation of plants which require fewer chemicals for pest management and fertilization are also pursued objectives. Thus, there is an urgent need to increase the limited genetic variability of cultivated rice in resistance/tolerance to biotic and abiotic stresses, using the gene pool available in *Oryza* wild species (Jena, 2010). Unfortunately, gene transfer from wild species into cultivated rice can be hampered because of cross compatibility barriers, restriction of chromosome pairing to homologous (identical) chromosomes and linkage drags (Jena, 2010). Some of these constraints could be overcome through genetic or biotechnological manipulation of meiosis (Benavente et al., 1996; Martinez-Perez and Moore, 2008) but these tasks require a deepening of our current knowledge on meiosis in cereals.

Meiosis is the central event in sexually reproducing organisms and is highly conserved in eukaryotes. In meiosis, gametes are generated through a single round of DNA replication followed by two successive rounds of chromosome segregation to halve the number of chromosomes. It takes place in specific cells, the so-called meiocytes, which switch from mitotic to meiotic divisions in a finely regulated and still poorly understood process. At the onset of meiosis, and more concretely at early prophase I (leptotene, pachytene, and zygotene), a dramatic reorganization of the nucleus including changes in chromosome morphology is known to occur (Page and Hawley, 2003). How homologous chromosomes recognize and pair are the least understood meiotic processes (Ronceret and Pawlowski, 2010). Different mechanisms have been reported to be involved in chromosome recognition such as those depending on chromatin structure (Prieto et al., 2004; Phillips and Dernburg, 2006; Ding et al., 2010), loci of high transcription rate (McKee, 1996; Wilson et al., 2005), specific non-coding RNAs (Ding et al., 2012), and cytoskeleton-driven chromosome movements (Ding et al., 2010; Labrador et al., 2013). In recent years, important studies have shed light into the genetic control and progression of meiosis in rice through the characterization of mutants altered in meiotic processes and/or sterile phenotypes (Nonomura et al., 2007, 2011; Yu et al., 2010; Che et al., 2011; Wang et al., 2012). In addition, with the growing application of the omics approaches, several transcriptomics analysis have contributed to the identification of genes involved in meiosis in plants (Chen et al., 2010; Kubo et al., 2013). Moreover, strong evidence indicating that proteomics approaches are effective in the identification of plant meiotic proteins has also been shown (Sánchez-Morán et al., 2005). To the best of our knowledge, the few proteomic studies on plant meiosis have been carried out using full anthers and focused on early microspore stages or stress induced changes using gel based 2-dimension electrophoresis (2-DE; Imin et al., 2001, 2006; Kerim et al., 2003; Woo et al., 2006; Phillips et al., 2008; Liu and Bennett, 2011). The use of full anthers has been reported to be insufficient for the enrichment of meiotic proteins, suggesting that an important effort isolating meiotic cells is required (Sánchez-Morán et al., 2005). So far, only meiocytes of *Brassica oleracea* L. have been previously isolated for 2-DE analysis (Sánchez-Morán et al., 2005). Unfortunately, limitations associated to the 2-DE method, the lower yield of protein from meiocytes extracts, and troubleshooting related to database identifications could hamper the detection of specific meiotic proteins.

In this study, a body of knowledge of the proteins involved in the earliest stages of rice meiosis, when homologous chromosomes recognize each other and associate correctly in pairs to recombine is provided. A protocol for collection and preservation of meiotic rice panicles and for the isolation of rice meiocytes has been developed to enrich protein extracts in meiosis-related proteins. The proteomes of rice meiocytes at each stage of prophase I (leptotene, zygotene, pachytene, diplotene, and diakinesis) have been characterized by nLC MS/MS, focusing on the proteins identified only at early prophase I stages (leptotene, zygotene, and pachytene) when chromosome recognition and pairing occur. Deciphering the proteins involved in early meiotic events could provide a valuable tool kit to manipulate chromosome associations and therefore, promote inter-specific recombination between chromosomes of cultivated rice and its wild relatives in breeding programs. In addition, the proteomics results described in this work can be extrapolated to other related species such as barley, wheat, rye, or maize, whose full genome sequences are not available yet.

## Materials and methods

### Plant material

Seeds of cultivated rice Nipponbare (*Oryza sativa* L. spp. *japonica*, AA, 2n = 2x = 24) were germinated for 10 days at 25°C on water-soaked filter paper. Germinated seeds were grown in 7 × 7 × 11 cm pots in a soil mix containing low pH loam, lime free horticultural sand and horticultural grit (3:2:0.5; vol:vol:vol). Plants were subsequently, cultivated in flooded trays in a growth chamber under 14 h photoperiod of fluorescent light at 360 μE m^−2^ s^−1^, at 28°C (day) and 24°C (night) and 70–95% relative humidity. After 2 weeks, plants were transplanted into bigger (10 × 10 × 11 cm) pots and kept there until flowering.

### Collection of rice panicles in meiosis and discrimination of the meiotic stage

Fresh young panicles were fixed in ethanol:acetic acid (3:1, v/v) in three rounds of 10 min under vacuum kept on ice (450 mmHg, vaccum pump R-400, Pobel, Madrid, Spain). Subsequently, the fixed panicles were stored at 4°C for up to 3 months.

**Figure 1:**
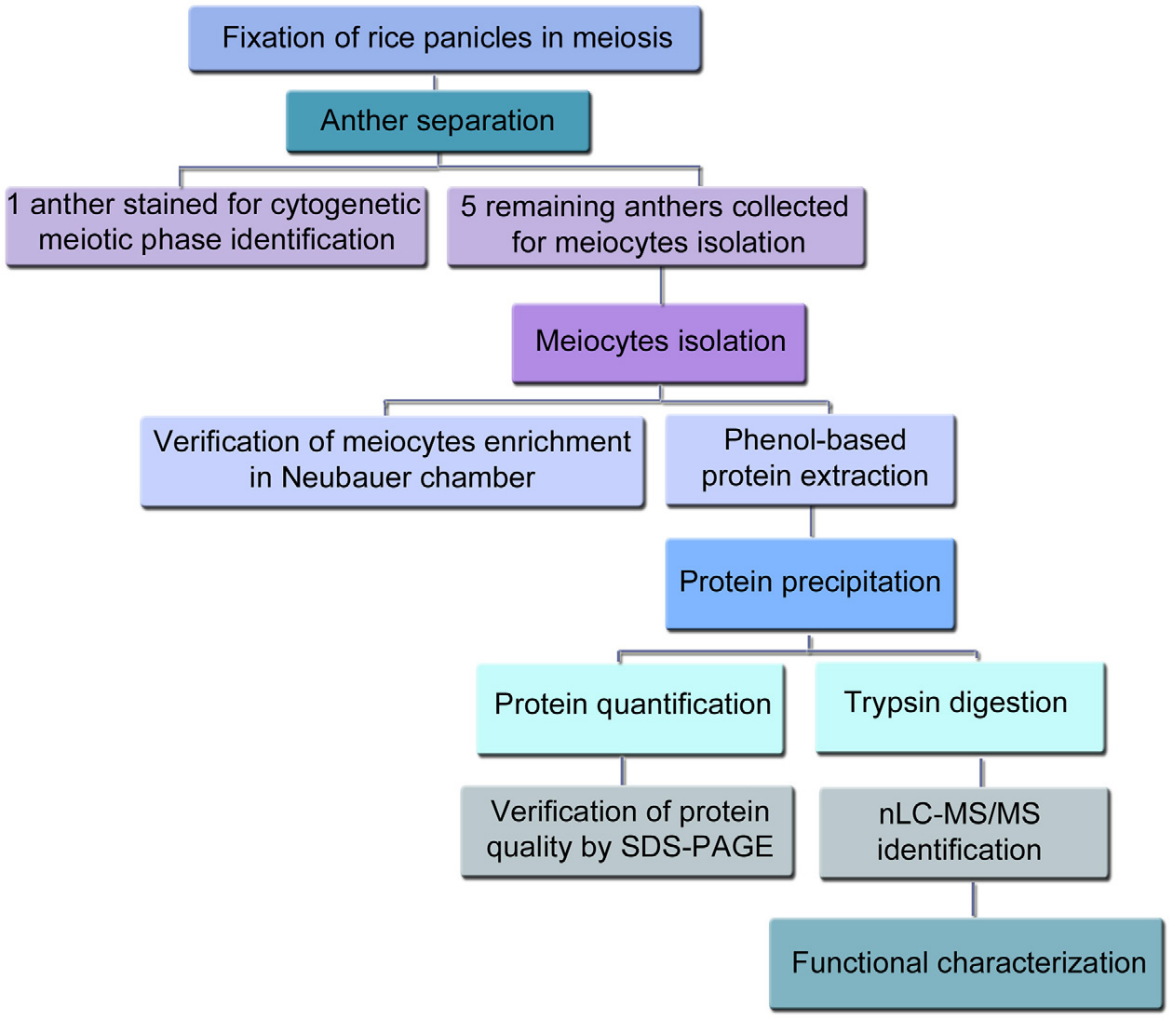
**Flow chart overview for the extraction of rice meiocytes in prophase I, the subsequent proteomics experiments and the data analysis**.

Ethanol and acetic acid were used as fixatives because they have been previously proved to be successfully employed in proteomic analysis (Ahram et al., 2003; De Souza et al., 2004; Milcheva et al., 2013) (Figure 1). Although the anther length is generally used as a criterion to determine the developmental stages of anthers in rice (Kerim et al., 2003; Itoh et al., 2005; Nonomura et al., 2011), an anther from each fixed flower was removed using fine forceps under a dissection microscope (Stemi 2000-C stereomicroscope, Carl Zeiss, Göttingen, Germany) equipped with a cold-light source to stage meiosis as much accurately as possible. Anthers were then stained in acetocarmine solution, squashed on ethanol-cleaned slides and checked under a PrimoStar light microscope (Carl Zeiss, Göttingen, Germany). The identification of the meiotic developmental stage was based on previous cytological descriptions of rice male meiosis (Chen et al., 2005; Itoh et al., 2005). Photographs were taken using an AxioCam ICc3 digital camera (Carl Zeiss, Göttingen, Germany) attached to the microscope.

### Anthers collection and meiocytes isolation

After the identification of the meiotic stage, the five remaining anthers of each flower were immediately collected in ice cold phosphate buffer saline (PBS) containing 1% protease inhibitor cocktail set VI (PI) (Calbiochem, Merck Chemicals Ltd. UK) (Figure 1). Although it has been previously described that there is synchrony among PMCs within an anther and among anthers within a single flower, and that this synchrony of male meiosis is thought to be established during premeiotic interphase (Nonomura et al., 2011), we have also checked and confirmed such synchrony by visualizing the meiotic stage of all the anthers in 10 different flowers (Figure S1). In addition and in order to take more synchronous anthers, only the main tillers of the rice plants were used in this experiment to isolate the anthers. For the isolation of the meiocytes the protocol previously described for isolation of *Brassica* meiocytes (Sánchez-Morán et al., 2005) was followed with some modifications. In brief, groups of 10 staged anthers were placed on 15 μl of PBS and 1% PI in a well of a multi-well slide (ICN Biomedicals Inc., Ohio, USA). Then, anthers were halved using two fine needles (0.5 ml BD Micro-Fine+ Insulin Syringes, 30G, 0.3 × 8 mm, BD Biosciences, Erembodegem, Belgium) under the stereomicroscope and gently squeezed to obtain a suspension of meiocytes, which was recovered in 1.5 ml tubes using a micropipette tip and kept on ice. Sample aliquots were loaded into a Neubauer chamber for checking successful meiocytes isolation/enrichment under the light microscope. Isolated meiocytes were stored a −80°C until protein extraction.

### Protein extraction and quantification

Proteins were extracted from pool of meiocytes isolated at each prophase I stage from a mix of anthers from at least 25 different rice plants, using a phenol isolation protocol (Tan et al., 2007) with some modifications. Considering the variability in each sample of meiocytes and the difficulty for obtaining enough amount of protein from isolated meiocytes at each stage of early meiosis, only one experiment was carried out at each meiotic stage. Frozen meiocytes were resuspended in phenol extraction buffer (0.9 M sucrose, 0.5 M Tris-HCl, 50 mM EDTA, 0.1 M KCl, Milli-Q water and freshly added 1% Triton X-100, 2% β-mercaptoethanol, and 1% protease inhibitor cocktail set VI (Calbiochem), pH 8) and homogenized on ice using Eppendorf micropestles. The solutions of meiocytes were sonicated with an ultrasonic homogenizer (Sonopuls HD2070, BANDELIN electronic, Berlin, Germany) during three cycles of 1 pulse of 20 s followed by 1 min on ice. Samples were then mixed with one volume of phenol solution equilibrated with 10 mM Tris HCl, pH 8.0, 1 mM EDTA (Sigma-Aldrich, St. Louis, MO), shaken for 1 min, incubated for 20 min in a tube rotator at 4°C and centrifuged at 18000 × *g* for 10 min at 4°C. The upper phenolic phase was collected and proteins were subsequently precipitated by adding five volumes of ice cold 0.1 M ammonium acetate and 13 mM DTT in methanol at −80°C for at least 2 h or overnight. A pellet of proteins was obtained by centrifugation at 20000 × *g* for 20 min at 4°C. Then, the pellet was washed once with ice cold 0.1 M ammonium acetate, 13 mM DTT in methanol and twice with 80% ice cold acetone. The washes consisted on the incubation of the resuspended pellet for 2 h at −20°C followed by 20 min centrifugation at 20000 × *g* for protein recovery. Finally, the pellet was air dried, dissolved in denaturing buffer containing 6 M urea, 50 mM ammonium bicarbonate pH 8 and stored at −80°C. Protein concentration was determined with the Pierce BCA Protein Assay Kit (Cultek SL, Madrid, Spain), using BSA as a standard according to manufacturer's instructions for the microplate procedure. Protein quality was checked by 1D-SDS-PAGE using Mini-Protean cell (Bio-Rad Laboratories, Alcobendas, Spain) and 12% Mini-PROTEAN® TGX™ precast polyacrylamide gels (Bio-Rad) stained with Coomassie Blue G250.

### Reverse phase-liquid chromatography RP-LC-MS/MS analysis

Protein extracts in 6 M urea and 50 mM ammonium bicarbonate pH 8 were reduced and alkylated. Disulfide bonds from cysteinyl residues were reduced with 10 mM DTT for 1 h at 37°C, and then thiol groups were alkylated with 50 mM iodoacetamide for 1 h at room temperature in the dark. Samples were diluted to reduce urea concentrations below 1.4 M and digested using sequencing grade trypsin (Promega, Madison, WI) overnight at 37°C in a trypsin/protein ratio of 1:5 (w/w). Digestion was stopped by the addition of 1% TFA. Then, the supernatants were dried down and desalted onto ZipTip C18 Pipette tips (EMD Millipore Corporation, Billerica, MA) until mass spectrometric analysis.

Desalted digested proteins were dried out, resuspended in 0.1% formic acid and analyzed by RP-LC-MS/MS in an Easy-nLC II system coupled to an ion trap LTQ-Orbitrap-Velos-Pro mass spectrometer (Thermo Fisher Scientific Inc., Waltham, MA). The peptides were concentrated (on-line) by reverse phase chromatography using a 0.1 × 20 mm C18 RP precolumn (Proxeon Biosystems, Odense, Denmark), and then separated using a 0.075 × 100 mm C18 RP column (Proxeon) operating at 0.3 μl/min.

Peptides were eluted in a 120-min gradient of 5–40% solvent B (solvent A: 0.1% formic acid in water, solvent B: 0.1% formic acid, 80% acetonitrile in water). ESI ionization was carried out using a Nano-bore emitters Stainless Steel ID 30 μm (Proxeon) interface. The Orbitrap resolution was set at 30.000. Peptides were detected in survey scans from 400 to 1600 amu (1 μscan), followed by 20 data dependent MS/MS scans (Top 20), using an isolation width of 2 u (in mass-to-charge ratio units), normalized collision energy of 35%, and dynamic exclusion mode applied during 30 s periods. Peptide identification from raw data was carried out using the SEQUEST algorithm (Proteome Discoverer 1.3, Thermo Scientific). Database search was performed against MSU Rice Genome Annotation Project Database ver. 7.0 (file: all.pep downloaded from http://rice.plantbiology.msu.edu/index.shtml; Ouyang et al., 2007). The following constraints were used for the searches: tryptic cleavage after Arg and Lys, up to two missed cleavage sites, and tolerances of 10 ppm for precursor ions and 0.8 Da for MS/MS fragment ions. Searches were performed allowing optional Met oxidation and Cys carbamidomethylation. Search against decoy database (integrated decoy approach) was performed using false discovery rate (FDR) < 0.01. Protein identification by nLC-MS/MS was carried out at the CBMSO protein chemistry facility, a member of ProteoRed network.

### Bioinformatics and functional analysis of identified proteins

The output accessions obtained with the Proteome Discoverer software were exported to Microsoft Excel for data analysis. The genome annotation from the MSU Rice Genome Annotation Project Database and Resource (http://rice.plantbiology.msu.edu/index.shtml; Ouyang et al., 2007) was used to match locus identifiers (accessions) with their putative functions. Firstly, a table containing information of all the proteins identified at the five meiotic stages analyzed was generated (Table S1). And secondly, a table containing the proteins identified at early prophase I stages (leptotene, zygotene, and pachytene, Table S2A) and another table with the proteins exclusively identified in Early Prophase I but not found at later stages (neither diplotene nor diakinesis, Table S2B) were created. For proteins which were only identified at early prophase I, hyperlinks of MSU Rice Genome Annotation Project and Rice Annotation Project (RAP; http://rapdb.dna.affrc.go.jp/; Tanaka et al., 2008) were manually attached. MSU annotation was converted into RAP annotation using the ID converter tool in RAP database. Gene onthology (GO) annotations, InterPro domains data and information about the putative *Arabidopsis* (http://www.arabidopsis.org) or *Poaceae* orthologous genes were used to classify the identified proteins according to their functions. If no functional annotation was found in these databases, putative conserved domains or functions were searched through an amino acid sequence blast (protein-protein BLAST) using BLASTP 2.2.27+. Similarly, MSU or RAP accessions were searched in UniProtKB for additional information. The mass spectrometry proteomics results have been deposited to the ProteomeXchange Consortium via the PRIDE partner repository with the PXD001058 dataset identifier.

## Results

### Identification and isolation of rice meiocytes at early meiotic stages and protein purification

**Figure 2:**
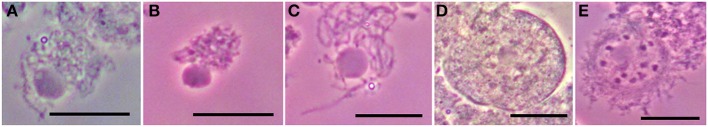
**Chromosome spreads of rice meiocytes in prophase I counterstained with carmine:acetic acid. (A)** Leptotene. **(B)** Zygotene. **(C)** Paquitene. **(D)** Diplotene. **(E)** Diakinesis. The nucleolus is visible during all prophase I. Bar = 20 μm.

**Figure 3:**
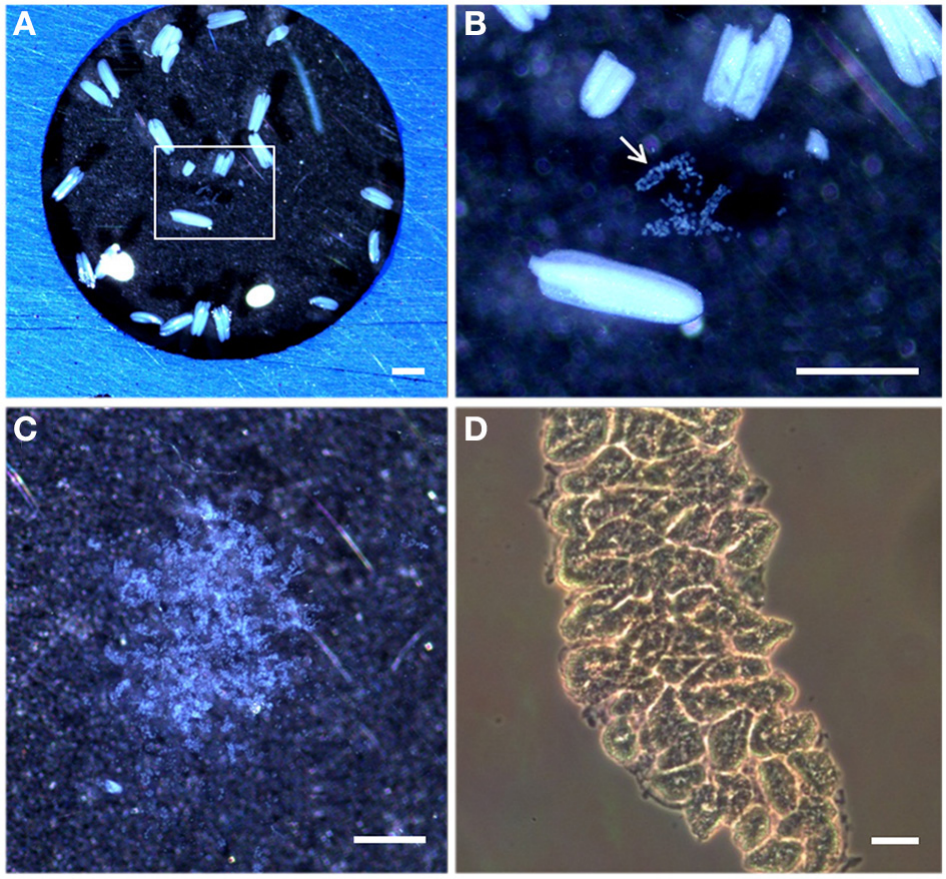
**Stereomicroscopy and light microscopy images of rice anthers and isolated meiocytes. (A)** Stereomicroscopy observations of rice anthers suspended in a solution PBS and 1% proteinases inhibitors on a multi-well slide. **(B)** Inset in **(A)** shows the meiocytes bag (arrowed) from a sectioned rice anther. **(C)** Rice isolated meiocytes under the stereomicroscope. **(D)** Light microscopy observations of rice isolated meiocytes in a Neubauer chamber. Bar represents 500 μm in **(A,B)**, and 20 μm in **(C,D)**.

In this work we developed a method to isolate rice meiocytes at different stages of early meiosis for the identification of the proteins involved in early meiotic events by nLC-MS/MS (summarized in Figure 1). First, fixed rice panicles were screened for the identification of the meiotic stage by checking one anther per flower. Rice anthers in prophase I stages were usually shorter than 800 μm and moreover, no significant differences in size were observed among the different stages of prophase I. Therefore, checking one anther per flower was necessary to unequivocally identify the meiotic stage (Figure 2). Since all the anthers in the same flower are synchronized, the remaining five anthers were immediately collected for the isolation of meiocytes (Figure 3). The number of flowers in a rice panicle at different stages of early meiosis varied from 1 to 6 at each stage of prophase I. Panicles from at least 25 rice plants (representing between 80 and 300 anthers, depending on the meiotic stage) were needed to obtain a minimum of 1.5 μg of protein necessary for each proteomic analysis. The protein yield ranged from 50 to 110 ng per flower. The isolation of meiocytes at diplotene was arduous since the number of flowers at this stage was scarce because of the rapid transition from diplotene to zygotene. Indeed, in other organisms such as *Saccharomyces cerevisiae*, the synaptonemal complex disassembly is so fast that diplotene is not even apparent (Dresser and Giroux, 1988). However, the protein yield per rice flower in diplotene was sufficient enough (1.5 μg) for nLC-MS/MS analysis.

**Figure 4:**
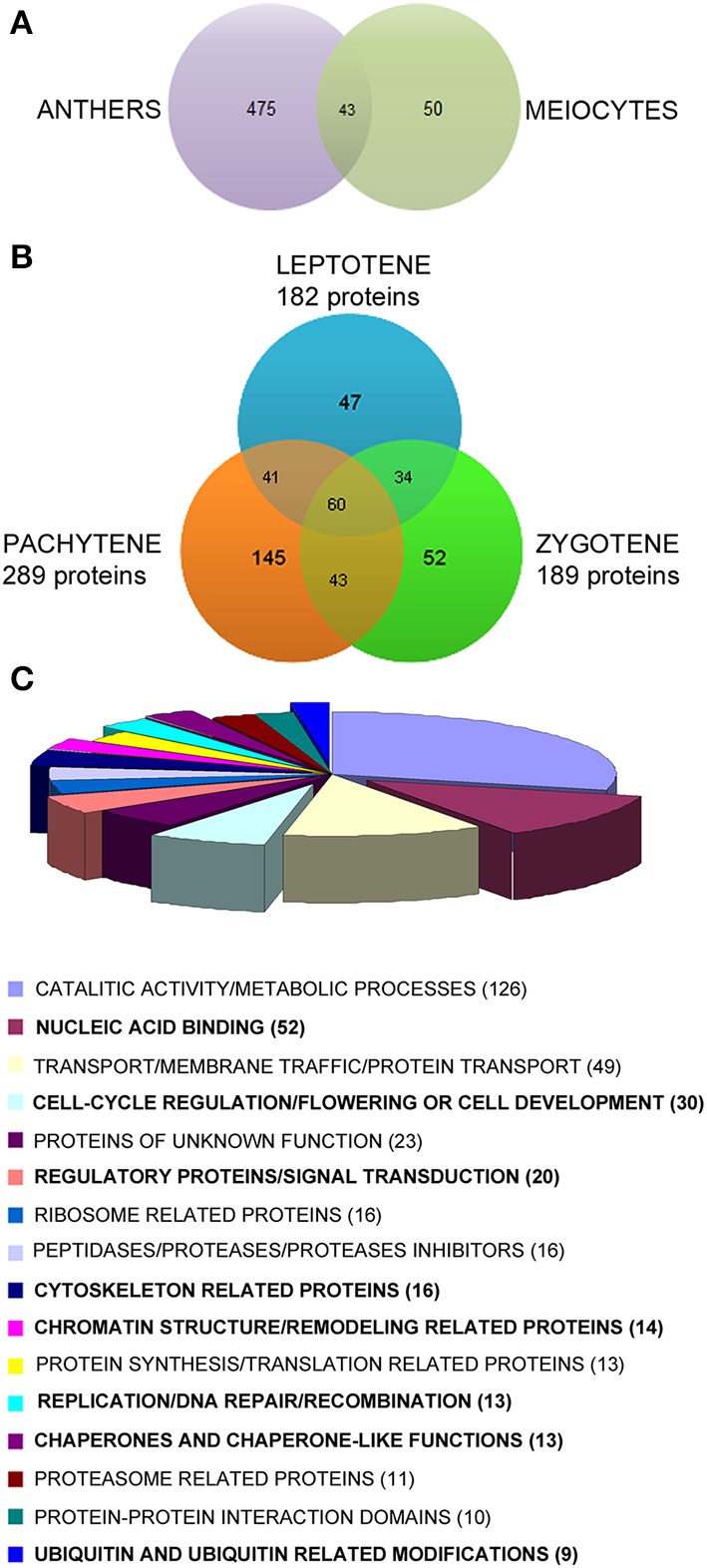
**(A)** Venn diagram of preliminary experiments comparing the proteins identified in a pull of full anthers and a pull of isolated meiocytes in meiosis (mix of different stages). **(B)**Venn diagram representing the proteins identified in rice meiocytes at early prophase I (leptotene, zygotene, and pachytene). The proteins identified at later stages (diplotene and diakinesis) were subtracted. Only peptides with 5–30 amino acids and a minimum of two peptides per protein were used for positive identification, and peptide FDR < 0.01. **(C)** Diagram of the biological functions associated to the 422 proteins identified only at early prophase I stages. The numbers of proteins associated to each function are in brackets.

The protein extraction protocol consisted on several rounds of extraction with a phenol-based buffer followed by ammonium acetate precipitation. The quality and the complexity of the extracted proteins were checked by 1D-SDS-PAGE prior to nLC-MS/MS (Figure S2). The complexity of the meiocyte extracts was different to that found in roots, whole anthers either in early or late meiosis and lemma (floral somatic tissue). It is worth to mention that the band pattern of whole anthers at different meiotic stages was rather similar (lanes 3 and 4), but significantly different to the meiocytes extract (lane 2), suggesting that the isolation of rice meiocytes led to the enrichment on meiosis-related proteins. Indeed, preliminary nLC-MS/MS analyses comparing the protein composition from whole anthers and isolated meiocytes revealed that only 8% of the proteins found in whole anthers were also found in isolated meiocytes (Figure 4A), suggesting that an important effort isolating meiotic cells is required in order to isolate proteins that are expressed in the meiocytes. Finally, since the isolation of rice meiocytes was critical and time-consuming, the individual protein extracts from each stage were not checked in a 1D-SDS-PAGE and used only for nLC-MS/MS analysis.

### Identification of proteins in rice meiocytes at early meiosis by nLC-MS/MS

To identify proteins that could play a role in early meiosis, protein extracts from suspensions of isolated rice meiocytes at each stage of prophase I were analyzed by nLC-MS/MS in independent runs. A high sensitive system of reverse-phase nLC coupled to a high resolution and outstanding mass accuracy of the LTQ-Orbitrap-Velos-Pro mass spectrometer was used to analyze each meiotic sample. The high mass accuracy of this equipment increases the speed and the confidence of protein identification in complex samples by minimizing false positives. Moreover, a high level of confidence was applied for protein identification to reduce false positives or misidentifications since only peptides with 5–30 amino acids and a minimum of two peptides per protein were required for positive identification. A FDR < 0.01 was also set. As a result, 1316 different proteins were identified from isolated rice meiocytes in all prophase I stages analyzed (Table S1).

The number of proteins identified was variable among the stages. Thus, 815 proteins were identified in leptotene, 787 in zygotene, 982 in pachytene, 139 in diplotene, and 891 in diakinesis. Although the number of proteins identified at diplotene was lower than in the other stages, this work was focused in earlier stages of prophase I, when chromosome pairing and recombination occur. Hence, the proteins identified in diplotene and diakinesis (late prophase I) were only used to establish a baseline protein profile of prophase I, that was then subtracted from the list of proteins identified at leptotene, zygotene, and pachytene (early prophase I; Table S2B).

At the earliest stages, when chromosomes recognize each other and pair (leptotene, zygotene, and pachytene), up to 1180 different proteins were identified (Table S2A), being 422 of them only identified in leptotene, zygotene, or pachytene but not in diplotene or diakinesis (Table S2B). These proteins were studied in detail since they could play a role in specific processes in early rice meiosis. Among them, 60 proteins were common to all three stages, while 47, 52, and 145 were only identified in leptotene, zygotene, and pachytene, respectively (Figure 4B).

### Functional classification of proteins identified in rice meiocytes only at early prophase I of meiosis (leptotene, zygotene, and pachytene)

The proteins identified only in rice meiocytes in leptotene, zygotene, or pachytene (422) were classified according to their putative biological function (Table S3). Thus, to get the most accurate classification, information from several databases (GO annotations, InterPro domains, KEGG, putative *Poaceae* or *Arabidopsis* orthologous), and additional p-blast searches, when no other information was available, was manually examined for all the proteins identified, and additionally complemented with information found in the literature (Table S3). A summary of the proteins grouped according to their biological functions is shown in Figure 4C. The most abundant proteins belonged to the category of proteins with catalytic activities and/or involved in metabolic processes (126 proteins), followed by proteins related to transport/membrane traffic/protein traffic (49) and nucleic acid binding proteins (41; Table S3).

A set of 167 proteins belonging to different functional groups were selected because, according to their function, they could be implicated in processes that occur at early meiosis, (highlighted in bold in Figure 4C and Table 1). Among them, three types of proteins have been intensively studied: (i) proteins related to chromatin structure and remodeling; (ii) proteins involved in regulation; and (iii) proteins related to the cytoskeleton. From these, and based on their putative function, we have selected 16 proteins as susceptible for further studies to check whether they play a role in chromosome pairing (Table 1, proteins marked with an asterisk).

**Table 1:** **Proteins identified in rice isolated meiocytes at early prophase I associated to putative functions involved in chromosome recognition**.

**Biological function**	**Molecular function**	**LEP**	**ZYG**	**PAC**	**MSU annotation**
**PROTEINS INVOLVED IN CHROMATIN STRUCTURE AND REMODELING**					
**Nucleosome components and chromatin structure**					
**Chromatin binding**					
(SMC2) chromosome segregation protein, putative, expressed	Protein-binding			2	LOC_Os01g67740.1
Histone deacetylase, putative, expressed	Catalytic activity			2	LOC_Os07g41090.1
Histone deacetylase, putative, expressed	Catalytic activity			2	LOC_Os08g25570.1
^*^ ***Chromatin-remodeling complex ATPase chain, putative, expressed (ATP-dependent chromatin-remodeling protein***	Catalytic activity		6		LOC_Os01g27040.1
^*^ ***SWIB/MDM2 domain containing protein, expressed (ATP-dependent chromatin-remodeling protein)***	Catalytic activity	4	2		LOC_Os02g03730.1
^*^ ***SWIB/MDM2 domain containing protein, expressed (ATP-dependent chromatin-remodeling protein)***	Catalytic activity	3			LOC_Os03g55310.1
^*^ ***PWWP domain containing protein, expressed***	Protein-binding		3		LOC_Os05g38810.1
**Nucleosome assembly related proteins**					
Core Histone H2A/H2B/H3/H4 domain containing protein, putative, expressed (H2A variant 3; H2A.3)	DNA-binding	3			LOC_Os03g53190.1
Histone H1, putative, expressed	DNA-binding	3		2	LOC_Os04g18090.1
Histone H3, putative, expressed (CenH3)	DNA-binding		2		LOC_Os05g41080.2
^*^ ***SET, putative, expressed (Nucleosome assembly protein, Nap1-related protein)***	DNA-binding	2	2	2	LOC_Os02g36710.1
***NAP domain containing protein, putative, expressed (Nucleosome assembly protein)***	DNA-binding			2	LOC_Os04g38620.1
**Centromeres associated**					
Tetratricopeptide repeat containing protein, putative, expressed (NASP-related)	Prot-binding		2	2	LOC_Os07g03070.1
CS domain containing protein, putative, expressed (nudC)	Prot-binding	3	3	5	LOC_Os06g12530.1
**Proteins involved in replication/DNA repair and recombination**					
DNA polymerase V, putative, expressed	DNA-binding		2		LOC_Os02g04270.1
Proliferating cell nuclear antigen (PCNA)—Putative DNA replicative polymerase clamp, expressed	DNA-binding	3		4	LOC_Os02g56130.1
MCM5—Putative minichromosome maintenance MCM complex subunit 5, expressed	DNA-binding	2	2		LOC_Os02g55410.2
RAD23 DNA repair protein, putative, expressed	DNA-binding		2		LOC_Os02g08300.2
RAD23 DNA repair protein, putative, expressed	DNA-binding		2	2	LOC_Os08g33340.2
Hydrolase, NUDIX family, domain containing protein, expressed	Catalytic activity		2	2	LOC_Os04g58900.1
3-5 exonuclease domain-containing protein, putative, expressed	Catalytic activity	2		2	LOC_Os01g47180.1
XPA-binding protein 2, putative, expressed	Protein-binding			3	LOC_Os07g44970.1
Phosphoesterase, putative, expressed	DNA-binding			2	LOC_Os01g53560.1
PPR repeat domain containing protein, putative, expressed	RNA-binding			2	LOC_Os01g67210.1
ruvB-like 2, putative, expressed (Reptin or TIP49b)	DNA-binding	4		3	LOC_Os06g08770.1
WD domain, G-beta repeat domain containing protein, expressed (REC14)	Protein-binding			3	LOC_Os11g43890.1
Basic helix-loop-helix, putative, expressed (APRATAXIN-like protein)	DNA-binding			2	LOC_Os03g18210.1
**Other DNA binding proteins**					
**DNA binding motifs**					
^*^ ***AT hook motif family protein, expressed (BRI1-KD interacting protein 135)***	DNA-binding		3		LOC_Os02g39920.1
^*^ ***AT hook motif family protein, expressed (BRI1-KD interacting protein 135)***	DNA-binding			2	LOC_Os04g42320.1
***AT hook motif domain containing protein, expressed***	DNA-binding		2		LOC_Os08g40150.1
***DNA-binding protein-related, putative, expressed (OB-fold nucleic acid binding domain containing protein)***	DNA-binding	2	2	2	LOC_Os01g13700.1
***DNA-binding protein-related, putative, expressed (OB-fold nucleic acid binding domain containing protein)***	DNA-binding		3	3	LOC_Os06g01370.1
***Transposon protein, putative, unclassified, expressed***	DNA-binding	2		2	LOC_Os03g52310.1
**RNA Polymerases**					
RNA polymerase Rpb1, domain 2 family protein, expressed	DNA-binding			2	LOC_Os02g05880.1
DNA-directed RNA polymerase subunit, putative, expressed	DNA-binding			3	LOC_Os09g02284.1
**Transcription regulation factors**					
Transcription initiation factor IIF, alpha subunit domain containing protein, expressed	DNA-binding			2	LOC_Os10g10990.3
CCR4-NOT transcription factor, putative, expressed (NOT2/NOT3/NOT5 family protein)	Nucleic acid binding		2	2	LOC_Os03g52594.5
PUR ALPHA-1, putative, expressed (PurA sequence specific DNA and RNA-binding protein)	Nucleic acid binding		2	2	LOC_Os01g15600.1
ZF-HD protein dimerization region containing protein, expressed	DNA-binding	2			LOC_Os12g10630.1
ZOS8-11—C2H2 zinc finger protein, expressed	DNA-binding		2		LOC_Os08g40560.2
Zinc finger helicase family protein, putative, expressed	Nucleic acid binding			3	LOC_Os01g15300.2
ZOS3-17—C2H2 zinc finger protein, expressed	DNA-binding		3	2	LOC_Os03g50850.2
SH2 motif, putative, expressed	RNA-binding	2	2		LOC_Os05g41510.1
**PROTEINS WITH KNOWN OR PUTATIVE FUNCTION IN REGULATION**					
**Cell-cycle regulation**					
^*^ ***Skp1 family, tetramerization domain containing protein, expressed (OSK27)***	Ubiquitin-related protein	3	2	4	LOC_Os07g43230.1
^*^ ***CDK5RAP3, putative, expressed (CDK5 activator-binding proteins)***	Protein binding			3	LOC_Os06g21560.1
^*^ ***E3 UFM1-protein ligase 1 (UFL1)***	Ubiquitin-related protein			2	LOC_Os05g02650.1
^*^ ***SIT4 phosphatase-associated protein family protein, expressed***	Catlytic activity	2			LOC_Os01g40340.3
Pachytene checkpoint protein 2 (AAA-type ATPase family protein, putative, expressed)	Catlytic activity		2		LOC_Os04g40290.1
Mitotic checkpoint protein, putative, expressed (putative Rae1)	RNA binding			2	LOC_Os01g44394.1
Split hand/foot malformation type 1, putative, expressed (Similar to DSS1)	Ubiquitin-related protein		2		LOC_Os01g16640.1
Protein kinase family protein, putative, expressed (SCY1-related protein kinase like)	RNA binding			2	LOC_Os01g42950.1
CWC15 homolog A, putative, expressed (Cwf15/Cwc15 cell cycle control protein family protein)	RNA processing	4	2	2	LOC_Os06g01700.1
Zinc finger C-x8-C-x5-C-x3-H type family protein, expressed	DNA-binding			2	LOC_Os04g57010.1
^*^ ***HECT-domain domain containing protein, expressed***	Ubiquitin-related protein		2		LOC_Os02g01170.1
Ubiquitin family domain containing protein, expressed	Ubiquitin-related protein	3	2		LOC_Os01g68950.1
Similar to FU (FUSED); MAPK KINASE 2-RELATED	Catlytic activity		2		LOC_Os12g24550.1
Prohibitin, putative, expressed	Protein binding	2	5		LOC_Os04g38900.2
Transposon protein, putative, CACTA, En/Spm sub-class, expressed	Protein binding			2	LOC_Os05g46780.1
**Development and flowering control**					
**Cell development regulation**					
GTP-binding protein, putative, expressed	RNA-binding	2	2	5	LOC_Os07g43470.1
GLTP domain containing protein, putative, expressed	Catalytic activity	2			LOC_Os03g57140.1
Similar to H0525E10.11 protein. (Exocyst complex component SEC5)	Ubiquitin-related protein			2	LOC_Os04g34450.1
Beta-catenin-like protein 1, putative, expressed	Nucleic acid-Protein bond			2	LOC_Os02g58460.1
DNA-binding TFAR19-related protein family protein	DNA-binding	4	3	3	LOC_Os05g47446.2
**Flower development/Flowering time**					
OsSPL13—SBP-box gene family member, expressed	DNA-binding	3		2	LOC_Os07g32170.1
Flowering time control protein FCA, putative, expressed	RNA-binding	2			LOC_Os09g03610.3
Glutamate–cysteine ligase, chloroplast precursor, putative, expressed	Catlytic activity	2			LOC_Os05g03820.3
Male sterility protein 2 (MS2), putative, expressed	Catlytic activity		2		LOC_Os08g44360.1
NLI interacting factor-like phosphatase, putative, expressed	Catlytic activity			2	LOC_Os05g43770.1
Polygalacturonase inhibitor 1 precursor, putative, expressed	Signal transduction	3	2	2	LOC_Os07g38130.1
Polygalacturonase inhibitor 1 precursor, putative, expressed	Signal transduction			2	LOC_Os09g31450.1
HEN1, putative, expressed (Methyltransferase type 12 domain containing protein)	RNA-binding			2	LOC_Os07g06970.1
KH domain containing protein, putative, expressed (FLK, flowering locus KH domain)	RNA-binding			2	LOC_Os03g42900.2
**Plant development**					
WD domain, G-beta repeat domain containing protein, expressed (Transducin/WD40 repeat-like superfamily protein)	Signal transduction			2	LOC_Os09g24260.1
**RNA processing/ Splicing factors/ RNA stabilization/ RNA recognition**					
**Splicing and/or RNA processing factors**					
Splicing factor 3 subunit 1, putative, expressed	RNA-binding			2	LOC_Os02g14780.2
Splicing factor, arginine/serine-rich, putative, expressed	Nucleic acid-binding	2			LOC_Os01g06290.4
Splicing factor, arginine/serine-rich 7, putative, expressed	Nucleic acid-binding			2	LOC_Os05g07000.1
RNA recognition motif containing protein, expressed	RNA-binding	2		3	LOC_Os06g08840.8
DEAD-box ATP-dependent RNA helicase, putative, expressed	RNA-binding	2	2		LOC_Os03g06220.1
Intron-binding protein aquarius, putative, expressed (DNA2/NAM7 helicase family)	RNA-binding	2		4	LOC_Os03g26960.1
RNA recognition motif containing protein, expressed (Arginine/serine-rich splicing factor RSP41)	RNA-binding		2		LOC_Os02g03040.4
RNA-binding motif protein, putative, expressed (Pre-mRNA-splicing factor SLT11)	RNA-binding			2	LOC_Os06g07350.1
WD domain, G-beta repeat domain containing protein, expressed (Pre-mRNA-splicing factor 19)	Protein binding		3		LOC_Os10g32880.1
Ribonuclease T2 family domain containing protein, expressed	RNA-binding	2			LOC_Os09g36700.1
LSM domain containing protein, expressed (Small nuclear ribonucleoprotein Sm D3)	RNA-binding	2			LOC_Os02g01250.1
Protein binding protein, putative, expressed (Pre-mRNA-processing protein 40A)	RNA-binding		2		LOC_Os01g34780.1
Proline-rich spliceosome-associated (PSP) domain containing protein, expressed	RNA-binding			3	LOC_Os02g58090.1
U5 small nuclear ribonucleoprotein 200 kDa helicase, putative, expressed	Nucleic acid-binding		2		LOC_Os02g01740.1
Cleavage and polyadenylation specificity factor subunit 5, putative, expressed	RNA-binding		2		LOC_Os04g58640.1
Transposon protein, putative, unclassified, expressed	Nucleic acid-binding	2	3		LOC_Os05g50624.2
**RNA recognition motifs**					
^*^ ***Transcription factor X1, putative, expressed (OXHS4)***	RNA-binding			2	LOC_Os02g19130.1
RNA recognition motif containing protein, expressed (Glycine-rich RNA-binding protein 7)	RNA-binding			2	LOC_Os07g08960.1
RNA recognition motif containing protein, putative, expressed	RNA-binding	2	3	2	LOC_Os05g13620.1
RNA recognition motif containing protein, putative, expressed	RNA-binding	3	2	4	LOC_Os07g33330.1
RNA recognition motif containing protein, putative, expressed	RNA-binding			2	LOC_Os06g02240.1
Expressed protein	RNA-binding	2			LOC_Os10g34370.1
PPR repeat domain containing protein, putative, expressed	RNA-binding		3	2	LOC_Os07g40800.1
Retrotransposon protein, putative, unclassified, expressed (DEAD-box ATP-dependent RNA helicase, putative)	RNA-binding		2	2	LOC_Os11g46240.1
Glycine-rich RNA-binding protein 7, putative, expressed	RNA-binding	3	2	3	LOC_Os03g61990.2
**Nucleus cytoplasm transport**					
**Nucleus-cytoplasm transport**					
Nucleoporin interacting component, putative, expressed	Protein binding		3	2	LOC_Os03g22690.1
OsNucAP3—Putative Nucleoporin Autopeptidase homolog, expressed	Protein binding			2	LOC_Os12g06890.1
Zinc finger family protein, putative, expressed (Similar to Testis expressed sequence 13A protein)	Binding			2	LOC_Os02g10920.4
Importin subunit beta, putative, expressed	Binding	2	3	2	LOC_Os12g38110.1
SRP40, C-terminal domain containing protein, expressed	Protein binding			3	LOC_Os12g41930.1
**RNA nuclear transport factors**					
Nuclear-pore anchor, putative, expressed	RNA-binding	2		2	LOC_Os02g50790.2
Nuclear-pore anchor, putative, expressed	RNA-binding	2	2	2	LOC_Os02g50799.1
RNA-binding protein-like, putative, expressed (Nuclear transport factor 2)	RNA-binding	2		2	LOC_Os02g29480.1
Nuclear transport factor 2, putative, expressed	RNA-binding	2	3	4	LOC_Os04g30430.1
Nucleoporin, putative, expressed	RNA-binding		2	2	LOC_Os03g12450.1
Nonsense-mediated mRNA decay protein 3, putative, expressed (NMD3 family protein, expressed)	RNA-binding	2	5	3	LOC_Os10g42320.1
**Other protein with putative regulatory functions**					
**Ubiquitination and related post-traslational modifications**					
E3 ubiquitin ligase, putative, expressed	Ubiquitin-related protein	2		2	LOC_Os01g49470.2
Ubiquitin family domain containing protein, expressed	Ubiquitin-related protein		2	2	LOC_Os02g10510.1
Ubiquitin family domain containing protein, expressed	Ubiquitin-related protein	2	2		LOC_Os03g03920.4
Ubiquitin-activating enzyme, putative, expressed	Ubiquitin-related protein		3	3	LOC_Os03g18380.1
Ubiquitin-conjugating enzyme, putative, expressed	Ubiquitin-related protein		2	2	LOC_Os12g41220.1
Ubiquitin carboxyl-terminal hydrolase, putative, expressed	Ubiquitin-related protein			2	LOC_Os08g41550.1
UBX domain-containing protein, putative, expressed	Ubiquitin-related protein			2	LOC_Os04g57520.1
UBX domain-containing protein, putative, expressed	Ubiquitin-related protein	4	2	3	LOC_Os08g43300.1
^*^ ***Defective in cullin neddylation (DCN) DCN1-like protein 2, putative, expressed***	Ubiquitin-related protein		3	2	LOC_Os06g12690.3
**Chaperones and chaperone-like functions**					
ATP-dependent Clp protease ATP-binding subunit clpA homolog CD4B,chloroplast precursor, putative, expressed	Catalytic activity	2		2	LOC_Os04g32560.2
ATP-dependent Clp protease ATP-binding subunit clpA homolog CD4B,chloroplast precursor, putative, expressed	Catalytic activity	2			LOC_Os11g16590.1
Chaperone protein clpB 1, putative, expressed	Catalytic activity	2	2		LOC_Os02g08490.1
Chaperonin, putative, expressed	Catalytic activity	2	3		LOC_Os03g25050.1
Chaperonin, putative, expressed	Catalytic activity	3	3		LOC_Os07g44740.1
Chaperonin, putative, expressed	Catalytic activity		2		LOC_Os09g26730.1
DnaK family protein, putative, expressed	Catalytic activity	3			LOC_Os03g50250.1
Dehydrogenase, putative, expressed	Catalytic activity		3		LOC_Os01g54940.1
hsp20/alpha crystallin family protein, putative, expressed	Catalytic activity			2	LOC_Os01g08860.1
Prefoldin, putative, expressed	Catalytic activity			2	LOC_Os12g37590.1
SCO1 protein homolog, mitochondrial precursor, putative, expressed	Catalytic activity		2		LOC_Os09g20430.1
Peptidyl-prolyl cis-trans isomerase, putative, expressed	Catalytic activity			2	LOC_Os01g18210.1
Peptidyl-prolyl cis-trans isomerase, putative, expressed	Catalytic activity		2	2	LOC_Os09g39780.2
**Proteins involved in signal-transduction/regulatory proteins**					
GTPase-activating protein, putative, expressed (Similar to ARF GAP-like zinc finger-containing protein ZIGA2)	Catalytic activity			2	LOC_Os03g63710.2
Zinc finger family protein, putative, expressed	Protein binding	2		2	LOC_Os08g36774.1
GCK domain containing protein	Signal transduction			3	LOC_Os03g44520.1
Ras-related protein, putative, expressed	Signal transduction	3			LOC_Os07g09680.1
Ras-related protein, putative, expressed	Signal transduction	2	2		LOC_Os10g23100.1
PB1 domain containing protein, expressed	Signal transduction		2	2	LOC_Os01g04650.1
Salt stress root protein RS1, putative, expressed	Protein binding	2		2	LOC_Os01g13210.1
WD domain, G-beta repeat domain containing protein, expressed	Catalytic activity	2		2	LOC_Os03g48090.1
PTEN, putative, expressed	Catalytic activity			3	LOC_Os12g21890.1
NB-ARC/LRR disease resistance protein, putative, expressed	Protein binding	2			LOC_Os03g14900.1
Zinc finger C-x8-C-x5-C-x3-H type family protein, expressed (BRI1-kinase domain-interacting protein 105)	Protein binding	2	2		LOC_Os02g06584.2
CBS domain containing membrane protein, putative, expressed (Similar to AKIN gamma)	Catalytic activity			2	LOC_Os01g40420.1
Copine, putative, expressed	Signal transduction			2	LOC_Os05g30970.1
phospholipase D, putative, expressed	Catalytic activity	2		3	LOC_Os01g07760.1
Serine/threonine protein phosphatase 5, putative, expressed	Catalytic activity			2	LOC_Os05g11550.2
Inactive receptor kinase At2g26730 precursor, putative, expressed	Catalytic activity		2	3	LOC_Os01g42294.1
Protein of unknown function DUF1296 domain containing protein, expressed	Nucleic acid-binding			2	LOC_Os06g10430.3
Universal stress protein domain containing protein, putative, expressed	Catalytic activity			2	LOC_Os01g57450.1
Universal stress protein domain containing protein, putative, expressed	Catalytic activity	2		4	LOC_Os05g42230.1
Zinc finger C-x8-C-x5-C-x3-H type family protein, expressed	Protein binding			3	LOC_Os01g61830.1
**CYTOSKELETON RELATED PROTEINS**					
**Actin related proteins**					
Actin, putative, expressed	Structural molecule activity	2	2	5	LOC_Os08g04280.2
Actin-depolymerizing factor, putative, expressed	Catalytic activity		2	2	LOC_Os03g56790.1
***ACBP4 (acyl-CoA binding protein 4), putative, expressed***	Protein binding	3	2	3	LOC_Os03g61930.2
***Kelch repeat protein, putative, expressed***	Protein binding			2	LOC_Os02g57690.1
Fimbrin-like protein 2, putative, expressed	Protein binding		2		LOC_Os01g33080.1
Fimbrin-like protein 2, putative, expressed	Protein binding		2		LOC_Os02g48740.1
Villin protein, putative, expressed	Protein binding	2			LOC_Os03g24220.1
Canopy homolog 2 precursor, putative, expressed	Binding			2	LOC_Os02g02524.1
WD domain, G-beta repeat domain containing protein, expressed	Catalytic activity			2	LOC_Os02g57220.1
Expressed protein	Structural molecule activity			2	LOC_Os04g54410.1
SH3 domain containing protein, expressed	Signal transduction		2		LOC_Os03g15900.2
**Microtubules related proteins**					
^*^ ***Microtubule associated protein, putative, expressed (MAP65-1a)***	Protein binding	6	3	4	LOC_Os06g20370.1
^*^ ***Targeting protein for Xklp2 (TPX2), putative, expressed***	Protein binding			2	LOC_Os06g40450.3
T-complex protein, putative, expressed (TCP-1)	Catalytic activity	2	3	3	LOC_Os02g22780.2
Lissencephaly type-1-like homology motif, putative, expressed (OsLIS-L1)	Protein binding	2		3	LOC_Os08g06480.1
Expressed protein	Catalytic activity	2			LOC_Os01g42770.1

For each protein MSU database description, molecular function, number of peptides identified at each early prophase I stage (LEP, leptotene; ZYG, zygotene; PAC, pachytene), and MSU database locus ID are provided. Proteins discussed in the text are in bold and italics. Proteins selected for further functional research are labeled with an asterisk.

Table 1 shows first the group of proteins involved in chromatin structure and remodeling (43), including: nucleosome components, chromatin binding proteins or proteins associated to centromeres (14); proteins involved in replication, DNA repair and recombination (13); and other DNA binding proteins (16). It is noteworthy that for most of the proteins, previous information about their specific function in meiosis was not available. Some examples of interesting proteins that have not been yet described as meiosis-related were: structural maintenance of chromosome-2 (SMC2), which is essential for chromosome condensation, the histone variants CenH3 and H2A.3 (*Arabidopsis* orthologous histone variant 2A.Z), and some proteins involved in DNA repair and recombination like three ATP-dependent chromatin-remodeling proteins and a protein with a Pro-Trp-Trp-Pro (PWWP) domain, two nucleosome assembly proteins (NAP) and six proteins containing DNA binding domains.

The second group of proteins that could be related to chromosome pairing includes proteins with a known or putative regulatory function (66; Table 1). This was the most diverse group among the three categories selected as putatively related to meiotic events, and was sub-classified into proteins involved in cell-cycle regulation, development and flowering control, RNA processing/recognition proteins, nucleus-cytoplasm transport, and other proteins with putative regulatory functions.

Finally, a group of cytoskeleton-related proteins (16) were analyzed since chromosome movements dependent on cytoskeleton might play an important role in chromosome recognition and stabilization and could act as a check-point mechanism for correct pairing. Proteins related to actin filaments (11) and to microtubules structures (5) were specifically identified in rice meiocytes in early meiosis (Table 1). For some of these proteins the available information about their specific roles related to cytoskeleton metabolism was very limited, and their classification in this group was based mainly on their annotated InterPro domains.

## Discussion

The main aim of this proteomic study was the identification of novel proteins involved in rice early meiosis, when chromosomes need to find a partner to pair and recombine before successfully segregate in metaphase I. Thus, new potential targets (proteins) to manipulate meiosis in general and chromosome pairing in particular were found. These proteins could be useful in plant breeding programs to facilitate chromosome recombination in inter-specific genetic crosses and to transfer, for example, valuable agronomical traits from wild relative species into cultivated rice. Therefore, a characterization of the rice meiocytes proteome at the earliest stages of meiosis (leptone, zygotene, and pachytene of prophase I) was carried out. Importantly and to the best of our knowledge, this is the first time that such a proteomic approach to meiosis has been developed in cereals. Moreover, rice proteomics studies can efficiently help to boost plant biology knowledge. In fact, the increasing information on the rice proteome can speed up the process of understanding not only biology of rice but also of other plants (Agrawal and Rakwal, 2011).

Despite of the enormous advances for protein analysis in high-resolution methods coupled to mass spectrometry and the availability of enriched rice databases (Kawahara et al., 2013; Sakai et al., 2013), proteomics analysis still have technical limitations for the whole identification of complex samples. Basically, these restraints are related to the resolution of nLC-MS/MS analysis and the availability of information in the databases and searching tools (Eng et al., 2011). In this work, identifications per each stage were carried out in independent runs of nLC-MS/MS, to increase the probability of identifying low-expressed proteins in each specific meiotic stage and to provide qualitative information about the presence of proteins among the first meiotic stages. A high number of proteins related to meiosis have been identified in this work, such as: a clathrin heavy chain protein, 15 DnaK family proteins, three RAD23 DNA repair proteins, one DNA-toposisomerase II, SKP1 and a suppressor of G2 allele of SKP1 (SGT1) (Madura and Prakash, 1990; Bai et al., 1996; Leroy et al., 1999; Zhao et al., 2003; Bansal et al., 2009; Hölzenspies et al., 2010) (Table S1). However, some others, which should be also expected to be present, were not found. Apart from the above-mentioned technical limitations, the absence of these proteins could be due to several reasons. For example, the characterization of many meiosis-related proteins has been previously carried out by the study of mutants, and moreover, most of the omics analysis have been based on mRNA (Chen et al., 2010; Kubo et al., 2013). In this respect, several studies have revealed poor correlations between the abundance of specific mRNAs and their corresponding proteins (reviewed in Rose et al., 2004; Alós et al., 2008).

Reducing sample complexity through single cell-type isolation or organelle isolation is a key point for a comprehensive and meaningful analysis in omics studies. The importance of isolating meiocytes for meiosis protein enrichment was previously reported in *Brassica* spp. where the 25% of the proteins identified in meiocytes enriched extracts were not identified using full anthers (Sánchez-Morán et al., 2005). In our experiments, the specific isolation of rice meiocytes allowed the enrichment on meiosis-related proteins at each early meiosis stage. In fact, preliminary analyses comparing the proteins identified in meiosis using whole rice anthers and isolated meiocytes revealed that, although the number of total proteins identified in isolated meiocytes was lower, most of them were not identified using complete rice anthers (Figure 4A). Therefore, the isolation of rice meiocytes was essential in this study, being the limiting step of the proteomic analysis mainly due to the low protein yield obtained, which required the collection of a high number of anthers per stage.

The mechanisms underlying homologous chromosomes recognition are mostly unknown, and this process remains even more elusive in polyploids, where several sets of similar genomes must search and pair with the correct partner. It is unlikely that DNA sequences are directly compared over the entire genome for chromosome recognition. Instead, the process of homologous recognition may involve chromosome-specific identifiers that can recognize homology at a first glance without comparing nucleotide sequences in detail, e.g., structural features specific to each chromosome (Ding et al., 2010). Moreover, early meiosis is also associated with changes in chromatin structure (Prieto et al., 2004; Colas et al., 2008). Therefore, chromatin structure and associated proteins (chromosome specific barcode), non-coding RNAs, active RNA transcription centers and cytoskeleton-dependent chromosome movements seem to play important roles in this process (Wilson et al., 2005; Ding et al., 2010, 2012; Labrador et al., 2013). Probably, all these mechanisms, or at least some of them, may function coordinately to provide successful chromosome pairing. In addition to the proteins that might be directly involved in these processes, regulatory proteins could also play an important role in coordinating events leading to pairing (Griffiths et al., 2006).

Chromatin structure has been widely reported as an important feature for chromosome pairing (Prieto et al., 2004; Colas et al., 2008) and a number of proteins involved in chromatin structure at early meiosis have been already described in plants (Hamant et al., 2006; Jenkins et al., 2008). In this study new proteins related to chromatin structure have been identified at early prophase I stages, namely: three ATP-dependent chromatin-remodeling proteins (LOC_Os01g27040.1, LOC_Os02g03730.1, LOC_Os03g55310.1), which could be involved in replication, DNA repair, recombination and transcription (Vignali et al., 2000; Struhl and Segal, 2013) and a PWWP domain containing protein (LOC_Os05g38810.1), which associates to methyl-transferases and regulate histone methylation (Wu et al., 2011; Qiu et al., 2012). It is worth to note that these four proteins were found only in leptotene or zygotene, suggesting specific functions related to nucleosome organization at these stages. Other interesting proteins found in this work are two NAP. One of them, SET (LOC_02g36710.1), whose implication in rice meiosis was not previously described, showed homology with *Arabidopsis* NAP1-related proteins which are known to be implicated in histone trafficking, nucleosome assembly and disassembly, and somatic homologous recombination in plants (Gao et al., 2012). In addition, among the proteins related to chromatin structure and remodeling, six uncharacterized proteins with DNA-binding motifs were identified. Two AT-hook motif family proteins, which are putative orthologous of the Brassinosteroid insensitive 1- kinase domain (BRI1-KD) interacting protein 135 (LOC_02g39920.1 and LOC_04g4230.1; Table 1), were annotated in HMM Panther as Androgen Induced Inhibitors of Proliferation (AS3)/PDS5-REL. A member of this family (PDS5) co-localizes with cohesin along meiotic chromosomes in yeast and the absence of this protein impairs homolog pairing leading to sister chromatids synapse (Jin et al., 2009). It is worth to mention that one of the AT-hook motif family proteins identified in this study (LOC_Os04g42320.1) showed 47% identity (35% coverage) with PDS5-like proteins from other species, which might suggest a role in chromosome pairing.

As expected, an important number of proteins involved in DNA repair and recombination has been identified in rice meiocytes. The contribution of homologous recombination to chromosome pairing varies among species and some recombination proteins may have dual roles in recombination and pairing, since similar mechanisms of chromatin structure modification might occur in both processes (Pawlowski and Cande, 2005; Ding et al., 2010). Therefore, we cannot rule out a putative role on chromosome pairing of the proteins involved in DNA repair identified in this study.

On the other hand, the fundamental role of regulatory proteins in coordinating events leading pairing has been reported in cereals. Thus, *ph1* mutants in wheat have an altered expression of *cdk2-like* genes that cause incorrect chromosome pairing and lead pairing between non-homologous chromosomes (Prieto et al., 2004; Griffiths et al., 2006). Interestingly enough, we have identified a SKP1 family protein (LOC_Os07g4323.1) which may function as a regulator of the cyclin A/Cdk2 complex (Bai et al., 1996). Therefore, this member of the SKP1 family could be associated to chromosome pairing in rice. Yeast and mammalian SKP1 regulate a wide variety of cellular events during the cell cycle (Okamoto et al., 2012). Whereas humans and yeast have a single SKP1 gene, many animal and plant species possess multiple SKP1 homologs, e.g., 21 in *Arabidopsis* (ASK or *Arabidopsis* SKP1-like) and 32 in rice (OSK or *Oryza sativa* SKP1-like; Kahloul et al., 2013). ASK1 participates in chromosome condensation, homolog synapsis and segregation in *Arabidopsis* (Yang et al., 2006), but so far, the role of rice OSK proteins has not been reported in meiosis. Interestingly enough five OSK proteins were also identified in prophase I in rice meiocytes, namely; OSK-1, OSK-20, OSK-22, OSK-26, and OSK-27 (LOC_Os07g43220.1, LOC_Os07g43230.1, LOC_Os07g43250, LOC_Os09g36830.1, LOC_Os11g26910.01). In fact, OSK-1 and OSK-20 could be the rice orthologous of *Arabidopsis* ASK-1 and ASK-2 (Kahloul et al., 2013). Hence, their presence in rice meiocytes at prophase I suggested their function in meiosis. Moreover, OSK-27 was identified only at early stages of prophase I, which might also indicate specific roles at early meiosis for this protein.

Another interesting protein identified in early meiosis that may play a regulatory role for rice cells entry into meiosis is CDK5RAP3, which is a CDK5 activator-binding protein (LOC_Os06g21560.1, Table 1). In mammals, this protein modulates CDK1-cyclin B1 function, promoting CDK1 activation and mitotic entry (Jiang et al., 2009). Therefore, CDK5RAP3 could play a similar role in rice cells entry into meiosis. Nevertheless, further studies are needed to elucidate its function. A putative CDK5RAP3 modified protein, the E3 UFM1-protein ligase 1 (UFL1, LOC_05g02650.1) that covalently attaches UFM1 modifier to cellular proteins, was also identified at early meiotic stages. The interaction between UFL1 and CDK5RAP3 prevents CDK5RAP3 from inhibiting cyclin D1 expression and is required for G1/S transition (Lemaire et al., 2011). Hence, the E3 UFM1-protein ligase 1 identified in this study could also be involved in cell-cycle progression in early rice meiosis.

Two new putative cell-cycle regulators, the SIT4 phosphatase-associated protein and a HECT-domain contacting protein (LOC_Os01g40340.3, LOC_Os02g01170.1), have been identified. SIT4 phosphatase-associated protein regulates SIT4 activity in *S. cerevisiae*, which is required for G1 cyclin transcription and bud formation (Luke et al., 1996). Moreover, cells without SIT4 are deficient in telomere-silencing ability (Hayashi et al., 2005). On the other hand, the *Arabidopsis* putative orthologous of the identified HECT domain-containing protein is involved in control of endoreplication cycles in trichomes (El Refy et al., 2003). Thus, these proteins could be involved in rice meiosis regulation and are, therefore, interesting candidates for future research.

RNA-binding proteins participate in meiosis regulation (Watanabe and Yamamoto, 1994; Harigaya et al., 2006; Nonomura et al., 2011) and have also been reported to be associated to non-coding RNAs which participate in chromosome recognition (Ding et al., 2012). In this study, 40 proteins with RNA-binding domains were identified at early prophase I stages, excluding those associated to ribosomes. These proteins might play a role during early meiosis although a specific function either in meiosis or cell-cycle has not been previously described for most of them. A protein with a RNA-recognition motif, OXHS4 (LOC-Os2g19130.1) could play specific functions in meiosis taking into account that its expression is restricted to young panicles, stamen, and pistil of rice plants (Qin et al., 2009).

Up to eight identified proteins, whose functions in meiosis have not been previously reported, were related to ubiquitination and ubiquitin-like modifiers. Ubiquitination targets proteins for proteasome degradation and is highly relevant in cell-cycle regulation. Thus, a central regulator of cyclin-dependent kinases activity is APC/C (Anaphase Promoting Complex/Cyclosome), a conserved multisubunit E3 ubiquitin ligase that triggers the degradation of multiple substrates (Cromer et al., 2012). Similarly, conformational changes in chromatin can be promoted by ubiquitination or ubiquitin-like modifications of synaptonemal complex components (Hooker and Roeder, 2006; Burger et al., 2013). Hence, the identification of a DCN1-like family protein (LOC_Os06g12690.3) at early stages may indicate a role of neddylation (conjugation of Nedd8 ubiquitin-like protein to a cullin protein) in regulation of early meiotic events in rice (Kurz et al., 2008). The importance of neddylation in cell cycle regulation was previously reported in *Caenorabditis elegans* where the neddylation pathways appear to be required for proper regulation of meiosis-mitosis transitions (Pintard et al., 2003).

Although at a first glance, proteins involved in the regulation of flower development and flowering time might not be considered as related to meiosis, alterations found for some of these proteins in flowering mutants might be a consequence of altered meiotic processes (Unte et al., 2003; Xing et al., 2011). Therefore, these proteins were included in this work and further analysis should be carried out to elucidate their role in meiosis.

Cytoskeleton-dependent chromosome movements might play an important role in chromosome recognition and stabilization and could act as a check-point mechanism for correct pairing (Ding et al., 2010; Labrador et al., 2013). The first step of chromosome pairing involves telomeres clustering at the onset of meiosis (Zickler and Kleckner, 1998). This movement is possible because telomeres are connected to cytoskeleton through SUN and KASH domain proteins which form a complex that expand nuclear envelope and connect telomeres to dynein protein motor on microtubules (Ding et al., 2010; Tiang et al., 2012). The importance of chromosome movement in pairing has been clearly shown in *C. elegans* and fission yeast (*Schizosaccharomyces pombe*, Ding et al., 2004, 2010; MacQueen et al., 2005; Phillips and Dernburg, 2006; Labrador et al., 2013). Then, although cytoskeleton-related proteins probably do not participate in chromosome recognition itself, chromosome movements are required for homologous chromosomes to associate and synapse. Actin filaments and microtubules are also known to have a role in chromosome movements during meiosis (Trelles-Sticken et al., 2005; Tiang et al., 2012). In this study, we showed a group of actin- and microtubule-related proteins that were identified only at early stages of prophase I in rice, and therefore might have important functions in chromosome movements during early meiosis. Among the microtubule related proteins (MAPs), two proteins, TPX2 and MAP65-1a, were highlighted (LOC_Os06g40450.3; LOC_Os06g20370.1; Table 1). In *Arabidopsis*, a rapid export of AtTPX2 from nucleus to cytoplasm during late G2 seems to be essential for plant pro-spindle assembly around the nucleus (Vos et al., 2008). In mitosis, during anaphase, this protein localizes to the spindle microtubules but not to the polar microtubules (Vos et al., 2008). On the other hand, MAP65-1a (microtubule-associated protein) could be the orthologous of the AtMAP65-2 according to MSU database and p-blast, which is a protein associated to cell cycle processes and seems to act as a strong stabilizer of microtubules (Li et al., 2009; Sasabe et al., 2011). A specific role of these two proteins in meiosis has not been reported yet.

Finally, proteins that might interact with actin filaments or be involved in their metabolism were identified in early meiosis and, to the best of our knowledge, a specific role of these proteins in cell-cycle has not been previously reported. Thus, further research would be needed to identify their roles in meiosis in general, or chromosome pairing in particular. Only for two of these proteins, ACBP4 and the Kelch-repeat protein (LOC_Os03g61930.2, LOC_Os02g57690.1), putative roles could be inferred. Thus, these two proteins with Kelch-type domains might have actin crosslinking functions and be associated to membrane according to database annotations. In fact, a protein containing a similar domain in *Drosophila* localizes to the actin-rich ring canals that connect the 15 nurse cells to the developing oocytes (Robinson and Cooley, 1997). Therefore, the proteins here identified could be involved in inter-meiocytes connections in rice during early meiosis. These connections are proposed to mediate the transport of metabolites and signaling molecules for synchronization of meiotic divisions in anthers (Mamun et al., 2005).

In summary, this work represents an important first step in applying proteomics to the study of the meiosis in cereals. For the first time a proteomic analysis has been carried out using rice isolated meiocytes at early prophase, when chromosomes recognize each other to associate correctly in pairs. The classification in functional groups allowed the identification of proteins involved in chromatin structure, nucleic acid binding, regulatory processes and cytoskeleton movements as putative candidates which could participate in early meiotic events. New proteins have been identified as candidates to manipulate meiosis. Future phenotypic characterization of transgenic overexpression or knock-down/out lines and/or mutants of many of these proteins will greatly contribute to understand their functions. In addition, this work provides important information to pave the way for a comprehensive analysis of the meiotic proteome of flowering plants. A better understanding of meiosis in crop species such as rice will help to target strategies to promote and manipulate meiotic recombination between non-homologous chromosomes from related species in breeding programs.

### Conflict of interest statement

The authors declare that the research was conducted in the absence of any commercial or financial relationships that could be construed as a potential conflict of interest.
